# Biomedical waste segregation compliance scoring system: to analyze, strengthen, monitor, and step up waste management in healthcare facilities

**DOI:** 10.11604/pamj.2023.45.163.35754

**Published:** 2023-08-16

**Authors:** Girisha Pindi, Swathi Suravaram, Siva Kandluri, Komali Donavalli

**Affiliations:** 1 Department of Microbiology and Infection Control, Employee State Insurance Corporation Super Speciality Hospital, Hyderabad, Telangana, India,; 2 Department of Microbiology, ESIC Medical College and Hospital, Hyderabad, Telangana, India

**Keywords:** Biomedical waste segregation, biomedical waste segregation compliance, biomedical waste monitoring, health care waste

## Abstract

**Introduction:**

segregation of biomedical waste (BMW) is the foremost and prime step for effective BMW management. This study was taken up to assess the BMW segregation compliance in patient care areas using a checklist-based scoring system to analyze the segregation compliance and establish feedback-based training programs.

**Methods:**

this study was conducted between January 2020 to December 2021 at a government tertiary care hospital in Hyderabad. The compliance was calculated by giving a score of one for each day, such that if there was no noncompliance (NC) the score was 100% for a given location at the end of the month. A score of minus one was given for each day a noncompliance was noted and transfigured into percentages. A score of 100% was considered good, and below 95% was considered an action point necessitating root cause analysis and training.

**Results:**

the BMW segregation compliance scores of the hospital for the year 2020 (96.5%) were compared with 2021 scores (97.5%). The outpatient department (OPD) and ICU had the poorest compliance rate of 93.7% and 93.6% respectively, compared to wards (96.2%). The most common factors influencing NC in BMW segregation were the joining of new staff, relocation, or new establishment of wards. The most common segregation error was found in the yellow disposal bags pertaining to the disposal of personal protective equipment.

**Conclusion:**

this easy and simple scoring system was established to improve the segregation compliance of BMW. End of each month an area wise compliance is easily made such that areas with low scores could be trained.

## Introduction

Biomedical waste (BMW) management has been a looming concern over the years. Though many countries have national hospital waste management guidelines in place adherence to the same has been a challenge. National BMW management guidelines have been published by the Govt. of India Ministry of Environment, Forest and climate change in 2016 [1]. Global baseline report (2019) by World Health Organization (WHO), Water, Sanitation, and Hygiene (WASH), and United Nations Children´s Fund, published that 1 in 3 healthcare facilities lacked systems for BMW segregation [2]. National-specific guidelines were added to the existing BMW guidelines to handle BMW generated during the care of COVID-19 patients [3].

The WHO´s “Blue Book” stated that only about 10-25% of waste generated is hazardous, making segregation an important step in preventing contamination of non-infectious from infectious waste which could pose a threat to the environment, increase the treatment and disposal costs and impose risks to health care professionals [4-6]. The impact of poor BMW management continues to persist in various countries despite guidelines being in place [7]. Effective segregation is pivotal and can alone ensure effective BMW management [8]. Hence, we have devised an easy-to-use scoring system to assess the bio-medical waste segregation compliance in patient care areas that aids us in understanding if effective BMW segregation is being practiced by the health care workers and retaliate with feedback-based training programs to strengthen BMW practices. The objective of this study is to use the BMW segregation compliance scoring system to monitor the BMW segregation practices in various patient care areas, to know the trends and pitfalls in BMW segregation, and to understand what are the common articles subjected to improper segregation such that training can be given to improve the practices.

## Methods

**Study design and setting:** this observational study was conducted to assess the BMW segregation practices in 15 patient care areas at a government super specialty tertiary care center in Hyderabad, India from January 2020 through December 2021 (Ethical approval no: ESICMC/SNR/IEC-F365/10-2021).

**Participants and study size:** the areas of the hospital where direct patient care was delivered or where patient diagnostic or treatment procedures were performed were considered as patient care areas and included in the study. Locations of the hospital where patient care was not rendered such as waiting rooms, walkways, canteens, kitchen, administrative departments, etc. were excluded from the study. Hence, the patient care areas in the hospital during the study period determined the sample size.

**Definitions:** the BMW definitions and regulations used in the study were as per the Government of India, BMW management, 2016 and 2018 (amended) guidelines [1,3,6]. The BMW disposal policy of the institute was revised and regularly updated and implemented as per the amendments.

**Data sources/measurement:** the BMW segregation was stringently monitored daily and evaluated as per a BMW segregation checklist (Table 1) by the infection control nurse (ICN) in coordination with designated hospital infection control (HIC) champions and nursing in-charges. The 15-point checklist was made sure to extensively cover various aspects pertaining to meticulous BMW segregation practices. If the area was a designated COVID care location, an additional 10-point checklist (Table 2) had to be followed in addition to the BMW segregation checklist provided [3]

**Table 1:** biomedical waste segregation compliance checklist

Patient care area assessed:	Date:		Assessed by:
Criteria	Response		Remarks
	Yes	No	
Designated BMW segregation area away from patients			
Instructions for segregation of BMW displayed in appropriate areas			
Availability of adequate color-coded (red, yellow, blue and white) bins for BMW and separate bins for general waste			
All the waste collection bins are covered with a lid?			
Are yellow and red bins lined with respective colored well fitted non-chlorinated plastic bags?			
White color sharp discard bins are puncture-proof, leak-proof, tamper-proof containers			
Blue-colored containers are puncture-proof and leak proof			
The BMW bins are labeled with a biohazard symbol or cytotoxic symbol along with the date and area of generation			
Segregation takes place at the point of generation			
BMW in the yellow bin is confined to human anatomical waste, soiled waste, discarded or expired medicine, chemical liquid waste, or chemical laboratory waste			
Separate yellow bins labeled with a cytotoxic symbol and biohazard symbol are used for the disposal of cytotoxic drugs			
BMW waste in red bins is confined to contaminated recyclable waste such as wastes generated from disposable items such as tubing, bottles, intravenous tubes and sets, catheters, urine bags, syringes (without needles and fixed needle syringes), and vacutainers with their needles cut) and gloves etc.			
BMW waste in white bins: confined to waste sharps (metals such as needles, syringes with fixed needles, needles from needle tip cutter or burner, scalpels, blades, or any other contaminated sharp object that may cause puncture and cuts)			
BMW waste in blue bins: confined to metallic implants and glassware (broken or discarded and contaminated glass including medicine vials and ampoules except those contaminated with cytotoxic wastes)			
Waste collection bags are not filled more than 3/4th of their capacity			
BMW: Biomedical waste; Satisfactory: +1; Unsatisfactory: -1	The score for the day:		Sign:

**Table 2:** checklist for biomedical waste segregation: COVID designated area

Patient care area assessed:	Date:		Assessed by:
Criteria	Response		Remarks
	Yes	No	
Separate color-coded bind labeled as “COVID-19 WASTE”			
Dedicated sanitation workers separately for biomedical waste and general solid waste			
The bins have foot-operated lids			
Bins lined with color-coded double-layered bags of adequate strength			
The inner and outer surfaces of the COVID-19 waste bins were cleaned with 1% hypochlorite daily			
General solid waste comprising of wrappers of medicines/syringes, fruit peel-offs, empty juice bottles or tetra packs, used water bottles, discarded papers, carton boxes of medicines, empty bottles of disinfectants, left-over food, disposable food plates, etc. should be collected separately as per SWM Rules, 2016			
Used mask (including Triple layer mask, N95 mask, etc.) head cover/cap, shoe cover, disposable linen gown, non-plastic or semi-plastic coverall in yellow bags			
Used PPEs such as goggles, face shield, splashproof apron, plastic coverall, hazmat suit, and nitrile gloves into a red bag			
Feces from COVID-19 confirmed in a patient, who is unable to use toilets and excreta collected in a diaper, must be treated as biomedical waste and should be placed in a yellow bag/container			
Appropriate PPE wore while handling COVID-19 BMW			
PPE: Personal protective equipment; BMW: Biomedical waste; SWM: Solid waste management rules; Satisfactory: +1; unsatisfactory: -1; not assessed: 0	The score for the day:		Sign:

**Variables:** both the checklists were made based on the national BMW management, 2016 and 2018 (amended) guidelines, updated as per revisions for COVID-19 [1,3,6]. The BMW that was discarded inappropriately and associated color-coded bins were documented. If a patient care area had more than one error in a defined calendar day pertaining either to multiple errors in various color-coded bins or one color-coded bin in various shifts, those were treated as one non-compliance for analysis.

**Missing data:** multiple observers made sure that the segregation practices were observed across their shifts and no non-compliances were missed.

**Quantitative variables:** the BMW segregation for a defined patient care area was considered satisfactory only if all the criteria provided in the checklist were marked as “YES” for the day and any breach was considered unsatisfactory. Bio-medical waste segregation compliance was calculated by giving a score of plus one (+1) for each day per location when found to be satisfactory and deducted by one for each day found to be unsatisfactory. The results were cached on the hospital computer database and the BMW segregation compliance rate was calculated at the end of the month. The BMW segregation compliance rate represented in percentage was calculated by dividing the total BMW compliance score obtained at the end of the month by the total number of days assessed. A score of 100% was considered as good compliance, and below 95% was considered an action point necessitating root cause analysis and training. The scores obtained in the year 2020 were compared with the scores in 2021.

**Bias:** to prevent variability in non-compliant BMW segregation photographs were taken of inappropriately segregated material by designated HIC champions and nursing in-charges and reported to the ICN. The utility of the BMW segregation compliance rate was assessed using two quality indicators- the number of needle stick injuries (NSIs) [9], reported as a result of segregation non-compliance and the amount of BMW generated per bed in kilograms [10]. The quality indicators used were compared with those of the previous years to see if this new method made any significant difference.

**Statistical methods:** the data was entered into Microsoft Excel and statistical analysis was performed using open -epiinfo. When the means of more than three groups were compared one way ANOVA was used and when two groups were compared T-test was used. A p-value of ≤0.05 was considered statistically significant.

## Results

**Figure 1:**
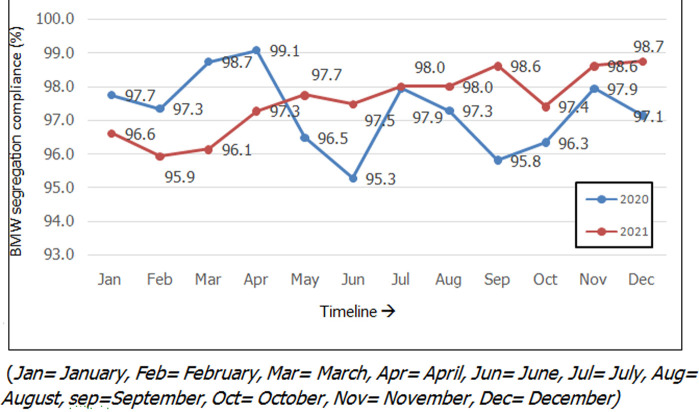
month-wise distribution of biomedical waste segregation compliance for the years 2020 and 2021

A total of 15 defined patient care areas were included to study the BMW segregation practices and were followed up through the study period using a checklist-based segregation compliance scoring system. The average BMW segregation compliance rates of the hospital for the years 2020 and 2021, respectively are 97.3% and 97.5% (p-value=0.4). The month-wise distribution of the scores is shown in Figure 1. Though there was no statistically significant difference in the overall BMW segregation practices between the years 2020 and 2021, it was found that the practices were found to be more consistent in 2021 with a standard deviation (SD) of 0.9 compared to 1.13 SD in the year 2020.

**Figure 2:**
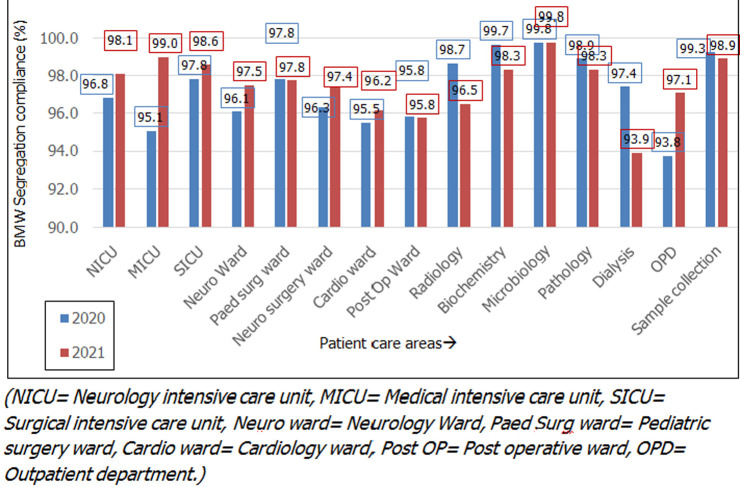
patient care area-wise distribution of biomedical waste segregation compliance

For the year 2020, the lowest scores were found in the months of June (95.3%) followed by September (95.8%) and October (96.3%). The noncompliance in the month of June 2020 (95.3%) was found to be statistically significant compared to the preceding months of May 2020 (96.5%) and April 2020 (99.1%) (p-value=0.02) and after corrective action, there was a significant improvement in the succeeding month of July (97.9%, p-value= 0.01). For the year 2021, the lowest scores were found in the months of February (95.9%) followed by March (96.1%). It was found that the BMW segregation compliance rate dropped consistently in the months of February and October for both years. Following corrective and preventive action (CAPA) sustained improvement was observed for a minimum period of 2 months. A collative comparison of the various patient care areas (Figure 2) has shown that the best segregation practices were followed in the departments of diagnostic medicine which included radiology, pathology, biochemistry, and microbiology (98.2%) compared to the intensive care units (ICU) (97.6%) and wards (96.6%). While the BMW segregation practices in the ICU´s improved from 96.6% in 2020 to 98.6% (p-value= 0.2) in 2021 they remained the same in the wards (2020=96.3% and 2021= 96.6%). In 2021, a significant improvement was observed in the medical ICU (2020=95.1% vs 2021=99%, p-value=0.04) and outpatient department (OPD) (2020=93.8% vs 2021=97.1%, p-value= 0.00001). A significant deterioration in the BMW segregation practices was observed in the dialysis unit (2020= 97.4% vs 2021 93.9%, p-value= 0.003) and department of radiology (2020=98.7% vs 2021=96.5%, p-value=0.02).

**Figure 3:**
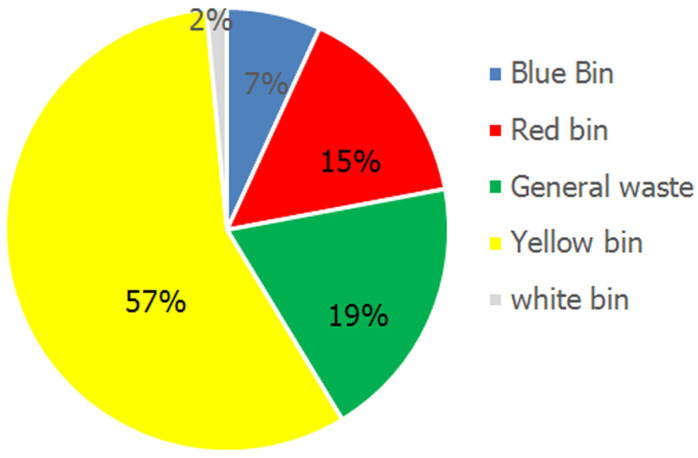
distribution of non-compliances found in various biomedical waste management bins

**Figure 4:**
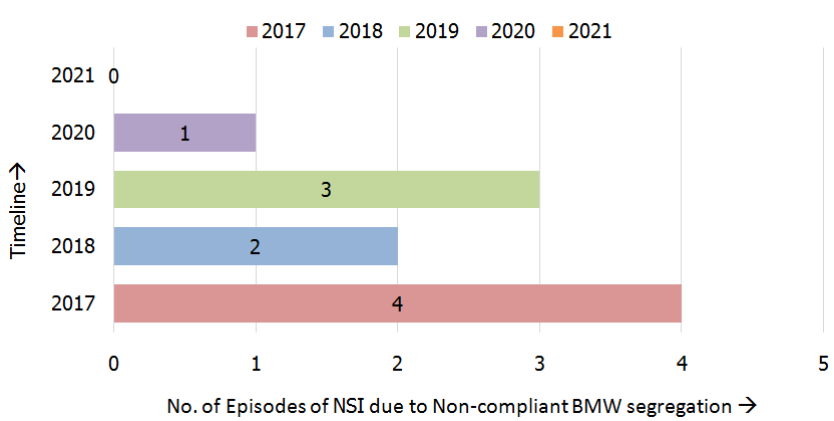
number of episodes of needle stick injuries (NSI) per year due to non-compliant biomedical waste segregation

**Figure 5:**
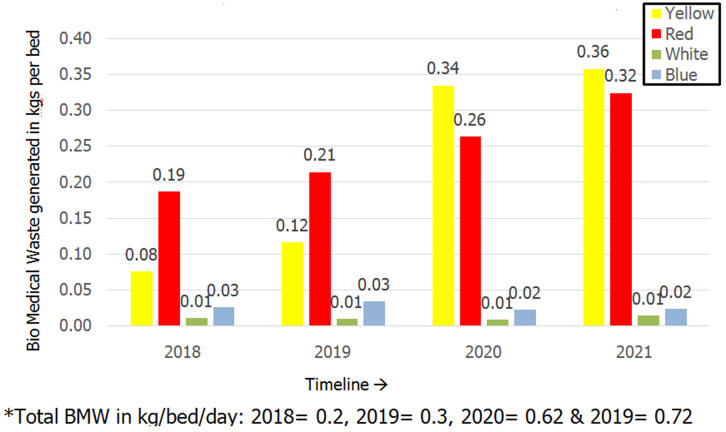
biomedical waste generated in kilograms per bed per year

It was noted that 57.6% of the segregation errors were found in the yellow BMW segregation bag followed by the general waste bag (19.4%) and red BMW bag (15.3%). The least noncompliance was found in the white puncture-proof container (2%) (Figure 3). The most common articles that were prone to segregation errors included plastic aprons and overalls (24.3%) followed by gloves (22.7%). The most common articles prone to segregation errors in the yellow bins were plastic aprons (33.9%) and gloves (31.1%). The most common items prone to segregation errors in the red and blue bins included soiled cotton (34.4%) and needles (58.3%) respectively. The most common BMW material found in the general waste bins were plastic aprons (25%) and gloves (25%) followed by surgical masks (19.4%). The trend of NSIs per year due to non-compliant BMW segregation practices reduced in the years 2020 and 2021 compared to the preceding three years as shown in Figure 4. In 2021 the hospital was upgraded from 150 bedded to a 200 bedded facility. The BMW generated in kilograms per bed per day was calculated and plotted on a graph in Figure 5. There was a steep rise in the amount of BMW generated from 0.37kgs/bed/day in 2019 to 0.62kgs/bed/day in 2021.

## Discussion

The awareness for better BMW management has increased over the years. Though it is a statutory requirement and stringent rules have been put forth by the Government, mismanagement of the BMW generated by healthcare facilities continues. This inefficiency could be due to lack of infrastructure, lack of knowledge, or due to lack of monitoring [11]. Adding to the need for appropriate BMW segregation, the advent of COVID-19 not only increased the amount of BMW generated and brought about changes in the composition but also the panic of getting infected reduced effective segregation adding new challenges to its management [12].

Emphasizing the importance of BMW segregation, a multivariate modeling study performed by the INCLEN program network across India found that having charts at the point of generation, availability of resources for segregation with access to appropriate personal protective equipment, accountability, and record maintenance were important predictors for a strengthened BMW management system [13]. In the present study, it was found that implementing a checklist-based scoring system for the calculation of BMW segregation compliance strengthened the practices and resulted in a sustained outcome (2020=97.3% and 2021=97.5%). A similar outcome based on daily and monthly BMW audits was found in the study done by Das *et al*. where the overall BMW management score was found to be 96.3% [14].

Complex studies based on multi-criteria decision analysis (MCDA) [15], weighing health care waste generated [16], and BMW deficiency index [5] were proposed to assess BMW management but the current study based on daily monitoring and compliance-based scoring is easy to implement and use. ICUs and OPD were found to have poor BMW compliance in the studies done by Das *et al*. [14] while the casualty was found to have the poorest BMW compliance in a study done by Mondal *et al*. [5]. Consistent with their findings the low BMW segregation scores in the present study were identified in ICUs (2020=97.6%) and high patient inflow areas such as dialysis units (2020= 97.4%) and OPDs (2020=93.8%). With focused training and monitoring BMW segregation compliance improved in the year 2021 with a 99% compliance rate in the medical ICU and 97.1% in the OPD. The massive fall in the BMW segregation rate in the months of May (96.5%) and June (95.3%) in 2020 was in sync with the COVID-19 peak when COVID-19 designated patient care areas were established. The consistent dip in the months of February and October in both years could be ascribed to the recruitment of new employees. Observations from questionnaire-based pre and post-training studies done by Hosny *et al*. [17] and Singh *et al*. [18] have shown that there was a statistically significant improvement in the knowledge of BMW management in health care workers following intervention but the effectiveness of the training was not mentioned.

In the current study, the interventions for improving BMW management included a comprehensive baseline assessment of the BMW practices, induction training to new staff via educational activities on the need for appropriate practices and current BMW guidelines, participatory training with emphasis on communication and inter cadre collaboration, customized audiovisual programs on BMW disposal, monthly feedback to heads of the departments with the segregation score and areas requiring improvement, reinforcing segregation of BMW at source using signages and spot training, scheduled awareness programs and quizzes. It was noted that a sustained response for a period of 2 months following an intervention for noncompliance was observed. From this, we could establish that quarterly training programs and continuous incidental training would be more appropriate for reinforcing BMW practices. To the best knowledge of the author, this is the first study to analyze which color-coded BMW bin was most prone to errors along with the type of article that was often disposed in the wrong bin. The study found that 57.6% of the segregation errors pertaining to BMW were found in the yellow bin contributed by plastic aprons (33.9%) and gloves (31.1%).

Pareto analysis in the study done by Dang *et al*. showed that 38.4% of NSIs were attributed to improper BMW segregation practices, and adequate training and infrastructure improvement reduced the incidence of NSIs from 0.05 NSI per health care woreker (HCW) to 0.03 NSI per HCW per year [9]. An improving trend in the reduction of NSIs was observed once monitoring of BMW segregation compliance was implemented in the current study with only 0.002 NSI per HCW reported in 2020 & no cases being reported in 2021 compared to 0.009 NSI per HCW in 2017. P.S. Thind *et al*. [19] in their study revealed that on average 3.41kg/day of BMW was generated by a COVID-19-infected patient in India and 50.44% of it was contributed by the waste disposed of in the yellow bin likewise in the present study there was a steep rise in the amount of BMW compared that generated in the pre COVID-19 era. Ferronato *et al*. [10] have proposed that assessment of the amount of healthcare waste could be used as a quality indicator for BMW management. In its application in the current study, it was found that the amount of BMW generated increased steeply from the pre-COVID era. Despite the increase in BMW generated in kgs/bed/day from 0.62 kg/bed/day in 2020 to 0.72 kg/bed/day in 2021 the number of NSI´s decreased owing to increased segregation compliance practices.

## Conclusion

Hence, in conclusion, it is to be noted that segregation plays a crucial role in BMW management. This scoring system is proposed for measuring BMW segregation compliance. Biomedical waste segregation compliance rate proposed in the present study is easy to apply and can be used for capacity building and strengthening of BMW segregation practices at various levels of health care system such as resource-poor settings, institutes without dedicated ICN´s, primary health centers, and rural or district level hospitals. Analysis of the non-compliances with respect to patient care areas, specific waste category, and disposed items will help trainers to focus on areas of concern. The study is limited by the fact that it is single centric and the efficacy of the scoring system can be reinforced by external validation. Though this scoring system is confined to only segregation it can be extrapolated to the other steps as well. Ensuring that the BMW guidelines are sustained and abided by, carries the same weight as having a comprehensive system in place, allocating responsibilities, training, and raising awareness of the risks of healthcare waste management.

### What is known about this topic

- *Biomedical waste management guidelines have been put forth by various countries;*
- *Improper BMW management has been a concern over the years and segregation plays a crucial role in BMW management*.

### What this study adds

- *The BMW segregation compliance scoring system is easy to use tool to monitor adherence to BMW management practices;*
- *The scoring system can be used for capacity building and strengthening of BMW segregation practices at various levels of health care system such as resource-poor settings, institutes without dedicated ICNs, Primary health centers, rural or district-level hospitals;*
- *Analysis of the non-compliances with respect to patient care areas, specific waste category, and disposed items will help trainers to focus on areas of concern and establish feedback training programs*.

## References

[1] Government of India Government of India Ministry of Environment. Forest And Climate Change Notification [Published in the Gazette of India, Extraordinary, Part II, Section 3, Sub-section (i)].

[2] World Health Organization & United Nations Children's Fund (UNICEF) WASH in health care facilities: global baseline report 2019.

[3] Central Pollution Control Board (2020). Guidelines for handling, treatment and disposal of waste generated during treatment, diagnosis, quarantine of COVID-19 patients. Central Pollution Control Board guidelines for COVID-19 waste management.

[4] Chartier Y (2014). Safe management of wastes from health-care activities. World Health Organization.

[5] Mondal R, Satyanarayana P (2017). A Study on Bio-Medical waste Segregation Monitoring in a tertiary Care Hospital at Andhra Pradesh. Asian Journal of Management.

[6] Central Pollution Control Board (2016). TOOLKIT Bio-Medical Waste Management Rules.

[7] Babanyara YY, Ibrahim DB, Garba T, Bogoro AG, Abubakar MY (2013). Poor Medical Waste Management (MWM) practices and its risks to human health and the environment: a literature review. Int J Environ Ealth Sci Eng.

[8] Shanthi Sree KS, Suvarna Latha A, Lakshmi Padmavathi PD, Bharathi (2019). Awareness on the bio-medical waste and its segregation methods. Int J develop Research.

[9] Dang N, Das S (2019). Reduction in Needle Stick Injury Rate among the Healthcare Workers in a Tertiary Care Hospital. International Journal of Research Foundation of Hospital and Healthcare Administration.

[10] Ferronato N, Ragazzi M, Torrez Elias MS, Gorritty Portillo MA, Guisbert Lizarazu EG, Torretta V (2020). Application of healthcare waste indicators for assessing infectious waste management in Bolivia. Waste Manag Res.

[11] Das SK, Biswas R (2016). Awareness and practice of biomedical waste management among healthcare providers in a Tertiary Care Hospital of West Bengal, India. International Journal of Medicine and Public Health.

[12] Capoor MR, Parida A (2021). Biomedical Waste and Solid Waste Management in the Time of COVID-19: A Comprehensive Review of the National and International Scenario and Guidelines. J Lab Physicians.

[13] Arora NK, Pillai RN, Maheshwari M, Arya S, Das Gupta R, Chaturvedi S (2014). Bio-medical waste management: situational analysis & predictors of performances in 25 districts across 20 Indian States. Indian Journal of Medical Research.

[14] Das P, Vikram K, Gaikwad U, Dani A, Nagarkar NM (2020). Quality assessment of biomedical waste management practices at a tertiary care centre by implementing daily and monthly audit. International Journal.

[15] Aung TS, Luan S, Xu Q (2019). Application of multi-criteria-decision approach for the analysis of medical waste management systems in Myanmar. Journal of Cleaner Production.

[16] Meleko A, Tesfaye T, Henok A (2018). Assessment of healthcare waste generation rate and its management system in health centers of bench Maji zone. Ethiop J Health Sci.

[17] Hosny G, Samir S, El-Sharkawy R (2018). An intervention significantly improve medical waste handling and management: A consequence of raising knowledge and practical skills of health care workers. Int J Health Sci (Qassim).

[18] Singh S, Dhillon BS, Nityanand AK, Kumar B, Bhattacharya S (2020). Effectiveness of a training program about bio-medical waste management on the knowledge and practices of health-care professionals at a tertiary care teaching institute of North India. J Educ Health Promot.

[19] Thind PS, Sareen A, Singh DD, Singh S, John S (2021). Compromising situation of India´s bio-medical waste incineration units during pandemic outbreak of COVID-19: Associated environmental-health impacts and mitigation measures. Environ Pollut.

